# Disability justice and collective access to labour and delivery care: a qualitative study

**DOI:** 10.1186/s12884-024-07036-3

**Published:** 2024-12-20

**Authors:** Meredith Evans, Lesley A. Tarasoff, Yona Lunsky, Kate Welsh, Laurie Proulx, Susan M. Havercamp, Susan L. Parish, Hilary K. Brown

**Affiliations:** 1 Department of Health and Society, University of Toronto Scarborough, 1265 Military Trail, Toronto, ON M1C 1A4 Canada; 2 Azrieli Adult Neurodevelopmental Centre, Centre for Addiction and Mental Health, 250 College Street, Toronto, ON M5T 1R8 Canada; 3 Nisonger Center, The Ohio State University, McCampbell Hall, 1581 Dodd Drive, Columbus, OH 43210-1257 USA; 4 Mercy University, Westchester Campus, 555 Broadway, Dobbs Ferry, NY 10522 USA; 5 Dalla Lana School of Public Health, University of Toronto, 155 College Street, Toronto, ON M5T 3M7 Canada

**Keywords:** Disability, Accessibility, Pregnancy, Perinatal care, Childbirth, Labour and delivery care, Obstetrics, Maternity care, Shared decision-making

## Abstract

**Background:**

People with disabilities experience perinatal health disparities. This qualitative study examines disabled people’s experiences of labour and delivery care from a disability justice lens.

**Methods:**

Semi-structured interviews were conducted between July 2019 and February 2020 with 31 women and transgender people aged 18–45 years with physical, sensory, and/or intellectual/developmental disabilities, who were living in in Ontario, Canada and had given birth in the previous five years.

**Results:**

People with disabilities described negative experiences of provider-driven, disrespectful, and discriminatory labour and delivery care that can be interpreted as examples of disability injustice and obstetric ableism. People with disabilities also described positive experiences of collaborative, respectful, and disability-affirming labour and delivery care that can be interpreted as examples of disability justice, facilitated by what feminist disability justice scholars and activists call collective access.

**Conclusions:**

Collective access to labour and delivery care can improve perinatal health care for people with disabilities and promote disability justice. Reimagining care-related decision-making as an interdependent, collaborative, respectful, and disability-affirming process shared between patients and providers can help to facilitate collective access to labour and delivery care.

**Supplementary Information:**

The online version contains supplementary material available at 10.1186/s12884-024-07036-3.

## Background

In North America, pregnancy among people with disabilities is common [1, 2], despite ableist misconceptions that disabled people are unlikely to experience pregnancy [3]. While healthy pregnancies are possible for people with disabilities [4], disabled people experience perinatal health disparities, including elevated risk of severe maternal morbidity and mortality [5, 6]; gestational diabetes, perinatal hypertension, and caesarean delivery [7]; perinatal emergency visits and hospital admissions [8, 9]; and newborn complications, including preterm birth and low birth weight [10]. Qualitative studies on disability and perinatal health care have documented inequitable care provision, with inaccessible health care facilities and unmet accommodation needs, poor communication from providers, inadequate provider knowledge of disability, and negative provider attitudes, including dismissiveness and unwillingness to provide assistance and support [11–18]. These disparities are perpetuated by the historical and ongoing reproductive oppression of people with disabilities, including through the denial of reproductive decision-making and forced sterilization [19–21].

This qualitative study contributes to the literature on disability and perinatal health by examining experiences of labour and delivery care. Labour and delivery is an especially critical phase of perinatal health care because of the sociocultural significance of childbirth as a rite of passage [22]. Feminist scholarship has identified childbirth as a transformative moment in the construction of gendered subjectivities and identities [22–27]. While white, middle-upper-class, cisgender, heterosexual, non-disabled women are met with gendered expectations to become mothers, disabled people are confronted with ableist, discriminatory, stigmatizing, and eugenic beliefs that they should not reproduce [3, 28, 29]. Childbirth is therefore an especially high-stakes moment for disabled people, with reproduction an implicit act of everyday resistance against stigmatizing cultural and medical beliefs that people with disabilities are unfit to be parents [29].

The medicalization of pregnancy and childbirth has conceptualized labour and delivery as a risky event that health care providers must manage [22, 30, 31]. Since the medical model conceptualizes disability as an individual health problem [32], childbirth is imagined as an especially risky event for disabled people; indeed, pregnant women with disabilities have reported being labelled high-risk despite this status not always being medically indicated [11, 33] and are more likely to have their birth medically managed than women without disabilities, including through elevated rates of surgical deliveries [7, 33]. Alternatively, a social perspective on disability questions why childbirth, motherhood, and parenting is presumed to exclude disabled people [3, 29, 34, 35].

Our article examines experiences of labour and delivery care from a social perspective, using disability justice theory to guide our analysis. Disability justice is an intersectional theoretical lens and social movement established by queer disabled women of colour that “aims to identify and change the root causes of injustice for people with disabilities—namely, the systems that do not prioritize or fail to consider the wholeness of those with disabilities” [36]. Perinatal health care is one such system that does not adequately account for the intersectional social dimensions of disability; providers lack disability-related training [37, 38] and services consistently fail to meet the accessibility needs of pregnant people with disability across all stages of perinatal care, from prenatal through postpartum periods [11–18]. A disability justice lens is especially relevant for identifying root causes of inequitable perinatal health care for people with disabilities beyond conventional rights-based frameworks of accessibility, often defined in terms of specific infrastructural or material requirements that need to be met [39]. Feminist disability scholars and disability justice activists argue that accessibility is a complex and ongoing process, one that is negotiated through social relationships [40–44]. They advocate for “collective access,” meaning forms of disability access facilitated by social relationships that move beyond privileging individual independence to honouring our collective *inter*dependence [45–50]. By analyzing the labour and delivery care experiences of people with disabilities and identifying moments of disability injustice and disability justice in care, this article asks: how is collective access to labour and delivery care thwarted and facilitated? How could collective access to labour and delivery care promote disability justice in childbirth?

## Methods

This article presents findings from the Disability and Pregnancy Study, which investigated the experiences of pregnant people with disabilities in Ontario, Canada. This qualitative study was overseen by an Advisory Committee that included people with disabilities, service providers, and decision-makers. Through regular meetings and consultations, the Advisory Committee provided invaluable input and advice during all stages of the research process, supporting research design (e.g., recruitment strategy, interview guides), study implementation (e.g., disseminating recruitment materials), data analysis (e.g., triangulating perspectives), and knowledge exchange (e.g., advising on recommendations for community reports). The core research team also included peer researchers in recognition of community expertise [51]. The protocol was approved by the Research Ethics Board at the University of Toronto (protocol number 35018). The study included 31 semi-structured interviews with 29 cisgender women and 2 transgender people (1 genderqueer/non-binary person and 1 transgender man) with physical, hearing, vision, and intellectual/developmental disabilities (IDD). We use the gender-inclusive language of pregnant people to reflect that pregnancy and birth are experienced by gender-diverse people [52] and because our study included both cisgender women and transgender participants.

Participants were recruited through purposeful sampling to increase diversity in disability type and social determinants of health, including race and locality. Recruitment materials were disseminated through the networks of the research team, Advisory Committee, and organizations that serve people with disabilities, parents, and/or pregnant people. Inclusion criteria included self-identifying as having a physical, sensory, and/or IDD, residing in Ontario, Canada, being 18 years of age or older, having given birth within the last five years, and being able to communicate in English or American Sign Language (ASL). Participants with IDD were asked additional screening questions before obtaining consent to ensure comprehension of voluntary participation and withdrawal [53].

Interviews were conducted in-person or by telephone or videoconference between July 2019 and February 2020, by a public health researcher and peer researchers who identify as disabled. Participants provided written consent. Support persons, if present, signed a confidentiality agreement. Interviews focused on participants’ most recent perinatal care experience. Questions were asked chronologically: participants were asked about the time before pregnancy, prenatal care, labor and delivery care, and early postpartum care. They were asked what types of care or services they accessed at each stage, if services met their needs, and what they recommended to improve care. Interviews were approximately one hour long and were audio-recorded. Participants received a 50 CAD gift card and a list of resources on mental health and social services upon interview completion. The research team later followed up with participants to ask how they were doing and if they wanted to add any further details. Interviews were transcribed, de-identified, and verified.

This article focuses on interview discussions of labour and delivery care experiences, inclusive of prenatal care related to labour and delivery (e.g., birth planning) and postnatal care immediately following birth (e.g., until hospital discharge). We analyzed interview data thematically, coding transcripts in NVivo 12 using abductive data analysis and a disability justice lens. In line with grounded theory, an abductive approach to analysis is like an inductive approach insofar as it is data-driven and prioritizes theory construction; however, while an inductive approach aims to be uninfluenced by existing theories, an abductive approach acknowledges the influence of existing theories. An abductive approach follows philosopher Charles S. Peirce’s use of “abduction” as a process of inference embedded within the act of meaning-making, where researchers are led away from old to new theory based on surprising data, as informed by the researchers’ positionalities and theoretical backgrounds [54]. This approach was chosen due to its capacity to generate new insights while drawing on existing theories. Our abductive analysis is influenced by the theories of disability justice and collective access; we used these existing theories to make sense of both negative and positive labour and delivery care experiences of people with disabilities in our study, interpreting such experiences as examples of disability injustice and justice. We aimed to construct new arguments about disability justice and collective access to labour and delivery care, grounded in our empirical data.

Before initiating coding, transcripts were reviewed in full; this process of data familiarization helped to contextualize labour and delivery care experiences within participants’ broader narratives of pregnancy and birth. Initial descriptive codes (e.g., birth plan, vaginal birth, caesarean, obstetric care, midwifery care) and interpretive codes (e.g., discrimination, provider-driven care, patient-centred care, feeling disrespected, feeling affirmed) were constructed to represent labour and delivery care experiences. Codes were then reviewed, refined, and organized thematically as examples of disability injustice and disability justice in labour and delivery care. To ensure rigor, validity, and credibility of data analysis [55], interviewers debriefed and took notes after interviews; negative cases or outliers were examined; and we triangulated sources, analysts, and perspectives (e.g., recruiting a heterogenous sample, independent coding by multiple analysts; regular consultations with the Advisory Committee). Themes were shared and discussed with co-authors to validate and refine interpretations. Our reporting adheres to the Standards for Reporting Qualitative Research guidelines [56]. All names for participants are pseudonyms.

## Results

Participants (*N* = 31) included people with physical disabilities (*n* = 14), sensory disabilities (*n* = 7), IDD (*n* = 6), and multiple disabilities (*n* = 4). Participants ranged in age from 18 to 45 years, with most participants aged 26 to 45 years (*n* = 28). Most identified as cisgender women (*n* = 29), heterosexual (*n* = 25), and white (*n* = 21); were married or in common-law partnerships (*n* = 22); had completed postsecondary education (*n* = 17); and resided in urban areas (*n* = 22). Participants had birthed between 1 and 4 children. In describing their most recent birth experiences, most participants (*n* = 29) gave birth in a hospital and had an obstetrician (OB) as their primary care provider (*n* = 20). About half of participants (*n* = 15) had caesarean deliveries, the majority of which were planned.

Participants described both positive and negative experiences of labour and delivery care. As outlined in Table 1, we organized these experiences into the two major themes of disability injustice and disability justice in care, and six sub-themes. We found that negative experiences of disability injustice in labour and delivery care were provider-driven, disrespectful, and discriminatory. We found that positive experiences of disability justice in labour and delivery care were collaborative, respectful, and disability-affirming.

**Table 1:** Themes and sub-themes

Disability injustice in labour and delivery care	Disability justice in labour and delivery care
Provider-driven care	Collaborative care
Disrespectful care	Respectful care
Discriminatory care	Disability-affirming care

### Disability injustice: provider-driven labour and delivery care

Participants described experiences of provider-driven labour and delivery care. Some providers managed childbirth by making care-related decisions unilaterally and excluding pregnant people with disabilities from decision-making related to the mode of delivery, birth plan, and everyday care. Jasmine, a young woman of colour^1^ with fetal alcohol spectrum disorder, recalled that her physician decided the mode of delivery without soliciting her input. She said, “The doctor said I was going to have a vaginal birth from the beginning. I was like, ‘Okay.’” Miriam, a woman of colour with multiple sclerosis (MS), had wanted a vaginal birth and, when a surgical birth was medically recommended, was upset that her OB “ignored” her surgical birth plan.

[Because the baby was breech], we were forced into a surgical birth [.] There’s a midwife [in another city who has permission to] do vaginal births for breeched babies [but in my city] midwives have to transfer care to the hospital [and transfer care to obstetrics]. I worked with my doula to put together a C-section birth plan, which I talked to [the OB] about and put in writing. I don’t think she even took the paper. I said, “I have it here and these are the things that are important to me.” She brushed it off.

I wanted the cord not to be cut for as long as possible. I wanted vaginal seeding^2^ which she said was ridiculous. I wanted my partner to tell me the gender of the baby [*becomes tearful*]. She just ignored it. – Miriam (physical disability)

Miriam wanted to have at least some of her wishes respected: “If not [a vaginal birth], then at least [follow] the birth plan.” Johanna, a white woman with a physical disability who uses a wheelchair, was excluded from decision-making in everyday care when she was moved by a nurse without her consent after waking up in the [intensive care unit] after her surgical birth.

I was kind of groggy. One of the nurses was like, “We’re going to turn you over,” and I was like, “No wait,” but I couldn’t express that. She was like, “Take a deep breath,” and I was like, “No, no, no,” and she just moved me. I was like, “Fuck.” I wish I had somebody in with me to say, “No, you can’t just move her.” – Johanna (physical disability).

### Disability injustice: disrespectful labour and delivery care

Participants recounted experiences of disrespectful labour and delivery care, including having providers dismiss their concerns, symptoms, and assessments of labour, make rude comments, and fail to offer disability supports or accommodations, including accessible communication. Miriam was concerned about the epidural procedure because of her MS; however, some providers dismissed her concerns and made insensitive remarks about her disability.

I was more worried about the epidural than the surgery. I spoke to my neurologist, she said that it would be fine and not to worry. That helped a lot. But I had to go to my specialist [to get this reassurance]. Everybody else was like, “I don’t really know what to say to that concern.” It was kind of shitty.

[Right before the surgical birth, the anesthesiologist] said, “Oh, you have MS? This is a big procedure, and you should know you will be vulnerable to an attack after this.”

I had been given zero choice. That was the mood and atmosphere which my child was delivered. My neurologist later called him an asshole, said that he had no business talking to me about a neurological condition and giving a prognosis. – Miriam (physical disability).

Luciana, a woman of colour woman with rheumatoid arthritis, had pain symptoms dismissed by providers when she had a flare-up immediately following her surgical birth.

I kept saying to the nurses, “I’m in pain.” They thought it was the C-section. I said, “No, that feels like a papercut.” They said, “Okay, we’ll give you oxycodone.” It was terrible. They didn’t believe me about the pain. I started having shortness of breath and my legs started to swell up. They said, “Oh that’s normal.” […].

I was in so much pain for everything. I couldn’t walk. And still, I didn’t get assessed for the pain. It was all about the baby, which is great, but what if there’s no mom to take care of the baby? They completely ignored my pain, my shortness of breath, every day they kept saying, “We’re going to discharge you tomorrow.” I was so fed up. I said, “Nobody’s listening to me here, I should go home.”

After I got home, I couldn’t walk, I was out of breath. I had to call the ambulance. I had heart failure, I had hypertension. I was a mess. – Luciana (physical disability).

Robyn, a white Deaf woman, was not believed by providers when she told them that her water broke. Heather, a white woman with a physical disability, said providers were dismissive toward her assessment of labour progression. She said, “I kept telling them—the baby’s coming now. They’re like, ‘Oh no, you’ve got lots of time.’ Yeah, she almost came on the elevator floor.”

Maria, a white woman with IDD, had a similar experience where her assessment of labour progression was dismissed by providers:

I fainted twice and the nurse was actually being rude to me. When I was telling her that the baby is coming out, she’s like, “Oh no, it’s okay. You can go to the washroom. I think you need to use the washroom.” After, she opened my legs and she seen the baby’s head was already out. I had a bad experience. – Maria (IDD).

Garima, a woman of colour with IDD, also recalled rude comments from providers during childbirth:

From the moment the paramedics picked me up [to go to the hospital to deliver], it didn’t feel like they cared that I was pregnant. It was not a great experience. I had some pretty rude nurses and a pretty rude doctor.

When I told them I was uncomfortable, they weren’t taking my concerns seriously. Then when I had the epidural, they were screaming at me near the end, they’re like, “Oh, come on. Push your body up.” I’m like, “I can’t. I can’t even move. Fuck off.” When I was giving birth, I started making noise. They were like, “Shut up.” – Garima (IDD).

Participants described a lack of support when seeking disability-related accommodations and needing to self-advocate. Shannon, a white Deaf woman, said the hospital was “resistant” to spending the funds to bring an ASL interpreter to her remote community for the birth. After threatening to make a human rights complaint, the hospital cooperated; however, Shannon said “it was a long process.” Robyn had an emergency surgical birth with a lack of communication from providers because the hospital did not provide ASL interpretation in a timely manner.

Three nurses and doctors grabbed me. They put me in a wheelchair, it looked like they took me to a surgery prep room. There was no communication.

I was in full shock. I was being shoved this way and that way, in pain. They didn’t say, “Would you mind rolling over?” They just rolled me over, testing one thing after another. No interpreter, nothing. I could see people speaking around me, but I didn’t know what they were saying. I felt alone, I felt stuck. I understood that we had to have a C-section right away, but I didn’t understand the *why* behind it. I was getting prepped for surgery, and I had no interpreter. […]

I was struggling. I was trying to tell them how I was feeling. I wasn’t feeling very good, but my hands were tied on each side of my body. They should leave one hand that’s free, especially for Deaf patients, because if not, I have no way of expressing myself. […]

We got the sense something was wrong. I could see my husband was crying and there was no interpreter around. The nurse said something to my husband. My husband was in total shock. He signed “dead.” […].

It was shock, complete shock. I wish I had access to the interpreter, when I arrived and throughout that whole process, so at least I could understand why the decision was made and why they were doing the things that they were doing. They never asked me how I felt, they never asked me if I knew what was happening.

Actually, the interpreter had arrived, but they wouldn’t let them in the room. They just made the call for me. That was very disempowering. – Robyn (Deaf/sensory disability).

### Disability injustice: discriminatory labour and delivery care

Participants with disabilities described experiences of labour and delivery care that were discriminatory, where providers were condescending, insensitive, and judgemental toward them as disabled pregnant people and parents, and dismissive of their capacities.^3^ Corey, a white autistic genderqueer/non-binary person with Ehlers-Danlos syndrome, said that some providers treated them “like a child,” making condescending assumptions that they were incapable. Siobhan, a white woman who is blind, described a patronizing experience with a nurse who assumed she could not walk:

I’m in the labour and delivery wing and they’re getting me ready [to be induced] but the nurse was very surprised and concerned that I was blind. She was reluctant to ever let me stand up off the bed. She said, “Call me if you have to go to the washroom, I have to physically support you over there and help you go to the washroom.” Which I found frustrating at the time because I can go to the washroom on my own. My mobility is totally fine. [.] She wrote it on my chart, “This patient cannot leave her bed.”

Later, we were walking around the delivery unit for a while to try and see if things would happen. My husband and I are speeding around the delivery unit and she’s like, “Ah! You can walk!” [laughs]. She was like, “You’re *amazing*!” – Siobhan (sensory disability).

After Robyn’s delivery, providers celebrated that her baby was hearing, insensitively perpetuating the ableist belief that being hearing is more desirable than being Deaf:

Her hearing test showed that she was hearing and so they got all excited and started clapping their hands. They were so happy, and they started hugging me. I’m like, “Okay?” I get it, because you are hearing and to you, this is good news. But you’re jumping up and down in front of a Deaf mom. It sends the message like, “Whew! Dodged that one! Your baby’s not Deaf like you are, mom!” It’s very disrespectful. They made sure to write in caps, “NOT DEAF.” – Robyn (Deaf/sensory disability).

Participants with IDD shared concerns about discriminatory and ableist provider judgements, specifically that providers would deem them incapable parents because of their disability and call child protection services to report their newborn at risk of harm. Maria did not disclose that she had an IDD to providers because she was afraid they would see her as an unfit parent. Similarly, Garima was reluctant to ask questions because she was concerned providers might infer that she had an IDD. Laura, a white woman also with IDD, described feeling “judged” by labour and delivery providers at her first birth, when providers reported her to child protection services. She was not permitted to leave the hospital with her baby until assessed by social workers from child protection services, and described feeling “pushed to the side” by labour and delivery care providers who did not provide her support, for example to wash or feed her baby.

### Disability justice: collaborative labour and delivery care

Participants described collaborative experiences of labour and delivery care that were characterized by shared decision-making between providers and patients. Providers approached decision-making as a collaborative process by sharing information, offering support, and soliciting, valuing, and appreciating patients’ input. Daisy, a woman of colour with a physical disability, described choosing her mode of delivery as an informed decision that was shared with her provider, who provided her with information:

I didn’t have a C-section. My doctor—our decision was to have me go into labour on my own. When he saw me, he asked, “Do you feel? Can you recognize all this signs?” They did a good part on this. So when I go into labour, I know. I watch for all these signs. – Daisy (physical disability).

Johanna also described choosing her mode of delivery as a shared decision with her OB, who provided her with information, solicited her input, and offered her support. Providers demonstrated good communication with each other, as well as with Johanna. She felt listened to, and that her input was valued and appreciated:

The first question was whether I was going to do a vaginal or a C-section. [The OB] was like, “I’m going to leave that up to you. These are the pros and cons to each of those options.” She said she was willing to try vaginal but that there was an increased risk. I did my own research about what other people have done and there are some people that did it vaginally but I was uncomfortable with the idea of having a vaginal birth and then having to do the C-section because [the vaginal birth plan] didn’t work. There’s so many people I knew who had a successful C-section. I don’t like the idea of not having it all planned so I decided to go that route. The [OB] was like, “That sounds good, I agree with your reasoning. [.]

 There was a care plan meeting organized, where everybody involved in my care, the nurses, [OB], anaesthesiologist, neurologist, everybody was going to come together for this one meeting and then [spouse] and I would be able to express what we were worried about and then we’d be able to stamp out the plan. That made me feel good because everybody was in the room and listening to me. It was the first time I’ve ever had that happen before and [OB] was really good for not letting the doctors talk over. – Johanna (physical disability).

### Disability justice: respectful labour and delivery care

When providers asked patients questions, listened to their concerns, provided explanations, and offered support by helping to advocate for necessary accommodations during childbirth, participants recounted feeling respected. Maria recalled a respectful childbirth experience, where providers asked questions and provided information:

[The OB] would always check up on me and she would ask me questions. She was giving me information, how it will be like when I give birth, which hospital, that stuff. [During labour] she was always asking me if I needed any more drugs for the pain. She was always right beside me until I gave birth. They were really caring. And helpful. – Maria (IDD).

Garima also described a respectful childbirth experience, where she felt listened to and supported by providers, who communicated information in a way that was accessible for her as a woman with IDD:

I felt like they respected me. I got great support, a great doctor. He let my husband cut the cord. He never talked to me like I was stupid but never talked to me in ways I never understood either. The nurses loved my baby, they loved me. They were very friendly, very nice. – Garima (IDD).

Robyn said she felt supported by the front desk staff who called for an ASL interpreter on her behalf:

The only reason why I got that interpreter fairly soon after [the birth] is because [the front desk staff person] had just learned Deaf culture in their sign language classes. It was very fortunate that that woman was there working. I just always think about that woman and how quickly she made those calls. If she hadn’t been there, I don’t know. – Robyn (Deaf/sensory disability).

Similarly, although Shannon had to self-advocate for ASL interpretation, she felt supported by her physician who helped to advocate for her right to an interpreter:

We actually had to get the doctor involved and advocate for us as well. He wrote a letter saying, “She needs to have this.” And it took us a few months of going back and forth and finally they were like, “okay” and they got an interpreter. […] I was lucky when the doctor jumped in and said, “She needs it,” and that’s probably how we got the interpreter. If the doctor didn’t do that, I probably would have fought with them. – Shannon (Deaf/sensory disability).

Jordan, a white transgender man with Ehlers-Danlos syndrome, described “very nice and very respectful” labour and delivery care providers who had adequate disability knowledge. The respectful care he experienced was enhanced by a nurse who asked about preferred names and pronouns upon being admitted to the labour and delivery unit; Jordan emphasized that it “would’ve been phenomenal” to have providers be similarly knowledgeable and respectful of gender identity throughout all stages of pregnancy.

### Disability justice: disability-affirming labour and delivery care

Participants described moments of disability-affirming care, when providers affirmed their capacities and adapted labour and delivery care in creative and collaborative ways. After being encouraged by providers and family members to terminate her pregnancy or give up the baby for adoption because of her disability, Jasmine chose to keep the baby and felt affirmed in her decision by a labour and delivery nurse who related her own experience:

Before I left the hospital, she said that she had a baby almost the same age as me and that was really comforting to know because she’s got a good job now and all. That means I can [too]. [It gave me] hope. – Jasmine (IDD).

After one of her children’s surgical births, Robyn’s OB honoured her request by holding her baby over the curtain to include her in the moment of birth as a Deaf woman:

I remember the doctor being very sweet. I made sure that the doctor understood—I actually got him to sign something to say that he would put the baby up over the curtain. I did not want to miss that, so the doctor was like, “Okay, I’ll do that.” – Robyn (Deaf/sensory disability).

Similarly, Siobhan’s OB adapted his care practices in creative and collaborative ways, such as facilitating a moment of skin-to-skin after delivery, providing verbal explanations of the newborn’s care, and describing the placenta after Siobhan asked to feel it:

As soon as she was born, she was going to be taken across to the other side of the room and given full resuscitation, breathing tubes and things. When she was born, [the OB] was so sweet. He put her up on my chest for 10 s and said, “Quick kiss and cuddle.” That was so special to me, to get that chance to hear her cry before she got the breathing tube, a little cuddle, to get that sensory experience, that time with her.

When they took her across the room, he stood right by my head and told me, “Now, they’re doing this, they’re doing this.” He described what was going on with my baby across the room. That was so nice, that meant the world to me. Because otherwise, it would be like, “They took my baby and I have no idea where she is and what’s going on.” So that was really, like, wow.

I asked if I could feel the placenta because I wanted to see what it was like. He let me feel the placenta, he was describing what it was like and saying, “Here are these lobes and these veins.” He took that extra 3 min to describe what was going on there. That stays with me. Taking that extra few minutes meant a huge deal. – Siobhan (sensory disability).

## Discussion

In this qualitative study, we applied a disability justice framework to examine people with disabilities’ experiences of labour and delivery care. We found that people with disabilities had negative experiences of provider-driven, disrespectful, and discriminatory labour and delivery care. We interpreted such experiences as exemplary of disability injustice, in which health care systems reduce pregnant people with disabilities to objects of management and providers fail to recognize them as whole persons with autonomy who are deserving of courtesy, consideration, and care. In contrast, we also found that people with disabilities had positive experiences of collaborative, respectful, and disability-affirming labour and delivery care. We interpreted these experiences as illustrative of disability justice through collective access, in which people with disabilities are seen as whole persons with autonomy and decision-making capacity, and care is approached by providers as an interdependent and shared process. Grounded in this empirical analysis, we argue that facilitating collective access to labour and delivery care is critical to the promotion of disability justice in perinatal health systems.

Our findings are consistent with previous studies that have documented women with disabilities being excluded from pregnancy care-related decision-making [14, 59, 60]; disrespected and dismissed by providers [12, 35, 60–62]; and not receiving accommodations [16, 35, 63]. We found that provider-driven care excluded pregnant people with disabilities from major care-related decisions (like the mode of delivery) as well as ostensibly minor care-related decisions (like when to cut the umbilical cord or move a patient). We found that participants felt disrespected when providers dismissed their concerns, symptoms, and assessments of labour; made rude comments; and failed to facilitate disability supports or accommodations, including accessible communication. Finally, we found that participants experienced discriminatory care that—through condescension, insensitivity, and judgement—implied disabled people were less capable of pregnancy and parenthood, and less valuable than non-disabled persons.

Pregnant people in general are subject to mistreatment during childbirth by providers as well as by perinatal health systems [64], and obstetric violence is a widespread phenomenon broadly defined as abuse and mistreatment during childbirth [65–68]. However, pregnant people with disabilities also experience obstetric ableism [69]. A unique form of obstetric violence that manifests when disabled people are devalued or dehumanized by perinatal health care systems, obstetric ableism manifests, for example, when pregnant people with disabilities are excluded from or encounter barriers to health care facilities, presumed to be high-risk, coerced to seek abortion or sterilization, excluded from reproductive decision-making, and assumed less capable of pregnancy, motherhood, and parenthood [69]. We suggest that the provider-driven, disrespectful, and discriminatory labour and delivery care reported in our study—which facilitates dehumanizing experiences that violate disabled people’s dignity and bodily autonomy—can be understood as not only examples of disability injustice but also examples of obstetric ableism.

Our finding are consistent with prior research on the importance of shared decision-making and respect in perinatal health care provision generally [70, 71] as well as specifically in the context of caring for pregnant people with disabilities [11, 62, 63]. Our findings are also similar to prior research on the importance of disability accommodations during childbirth [11, 12]. Building on previous research that has pointed to the importance of inter-provider and inter-disciplinary collaboration when caring for disabled pregnant women [11, 72], we argue that provider-patient collaboration, mutual respect, and affirming the capacities of people with disabilities are techniques that facilitate collective access to care and promote disability justice. We found that providers collaborated with participants in shared decision-making by soliciting, valuing, and appreciating patients’ input, sharing information, and offering support to the process (such as by encouraging questions and allowing time for reflection); respected participants by asking them questions, listening to their concerns, providing explanations, and offering support (such as by advocating for necessary accommodations); and provided disability-affirming care by affirming patient capacities and adapting care with patients in creative and collaborative ways. Through collaborative, respectful, and disability-affirming techniques of care, providers can better treat people with disabilities as whole persons. These techniques could enhances possibilities for perinatal health care providers and pregnant people with disabilities to work collectively to reimagine labour and delivery care as an interdependent and shared process.

Previous studies on the experiences of pregnant women with disabilities have called for improving patient choice as means of patient empowerment [14, 59, 60]. An emphasis on patient choice for all pregnant people with disabilities is also highlighted by the guidelines for perinatal and maternity care in Canada and the United States [73, 74]. The American Academy of Pediatrics and American College of Obstetricians and Gynecologists [73] state that providers should “strive to engage the family as co-providers and decision-making partners” by offering “real choices” and “respecting the choices” of pregnant women and their families. The Public Health Agency of Canada [74] affirms that pregnant women “are the primary decision makers about their own care” and should be “supported in the process of continued informed choice,” since family-centered maternity care “involves choices” and is “about increasing the participation of women and their families in the decision-making process concerning their pregnancy, birth, and early postpartum experiences.” As these guidelines suggest, patient choice is situated as be an intrinsic good in perinatal health care, ostensibly affording pregnant people autonomy and liberating them from passivity in their own care. However, deferring decision-making onto a patient can lead to poor care—the burden of responsibility for good or bad outcomes is placed on the patient rather than shared with the provider [75]. In the context of reproductive health care, individualized choice is constrained and limited in its capacity to afford autonomy and empowerment [76, 77]; furthermore, it erases the complexities of choice in reproductive health care, including the history of informed choice in midwifery as a relational care practice entangled with feminist politics [78]. In addition to the limitations of individualized choice in perinatal care more generally, we add that normative ideas about choice perpetrate obstetric ableism, insofar as only some non-disabled individuals are perceived to have the capacity to make independent choices about their care while other disabled people are not.

As people with disabilities’ labour and delivery care experiences illustrate, shared decision-making can be collaborative, respectful, and disability-affirming, and can facilitate collective access to care as an interdependent process. For example, Johanna’s decision to proceed with a surgical birth was not made independently but rather shaped by the information that her OB provided and was contingent on her OB’s support. Having her input valued by providers made Johanna feel respected and affirmed. During childbirth, especially for people with disabilities who are more likely to have their pregnancies deemed high-risk, complications can preclude opportunities for individual choice in labour and delivery care, such as for Miriam whose surgical birth was medically recommended. Instead of making people like Miriam feel “ignored” and devalued, providers can facilitate decision-making as a shared process through various techniques—soliciting, valuing, and appreciating patients’ input, sharing information, asking questions, listening to their concerns, providing explanations, offering support, advocating for accommodations, affirming patient capacities, and creatively adapting care practices in collaboration with patients. Facilitating collective access through shared decision-making and promoting labour and delivery care that is collaborative, respectful, and disability-affirming can disrupt assumptions of medical risk that position pregnant people with disabilities as objects of management and mitigate obstetric ableism in perinatal health care.

Shared decision-making can lead to better health outcomes [79] and is considered an important aspect of respectful, good quality labour and delivery care for all pregnant people [80–83]. The experiences of provider-driven labour and delivery care reported in our study demonstrate that people with disabilities can be denied opportunities for shared decision-making. Provider-driven labour and delivery care can be disrespectful and discriminatory to disabled pregnant people, discounting their autonomy as persons with decision-making capacity and promoting the medical management of childbirth. Since disabled parents are too often presumed to be incompetent [84] and incapable of making decisions [85], and pregnant people with disabilities are presumed to be high-risk, regardless of medical indication, and subject to increased medical interventions [7, 11, 33], shared decision-making is especially important for resisting systemic forms of disability injustice and obstetric ableism. Shared decision-making is a form of collaborative, respectful, and disability-affirming care that can facilitate collective access to perinatal health care for people with disabilities and promote disability justice.

Disability justice emphasizes that disability is an intersectional experience, and that disability is inseparable from other social dimensions of experience such as race, gender, sexuality, and class. Our findings are limited insofar as participants in our study were mostly white, cisgender women with disabilities from urban and suburban areas, and our study did not directly investigate how disability intersects with other domains of experience (gender, sexuality, race, class, locality, etc.) to shape pregnancy experiences.

Previous studies have highlighted that systemically marginalized communities including Black American women are less likely than white women to experience shared decision-making during labour and delivery care [86]. While our study included mostly white people with disabilities, we understand the negative labour and delivery care experiences of racialized people with disabilities in our study (e.g., Jasmine, Miriam, Luciana, and Garima) to be shaped by both obstetric ableism and obstetric racism [87]. Facilitating collective access to labour and delivery care as a collaborative process through shared decision-making is therefore especially important for perinatal health care that is committed to combating both ableism and racism, as well as other intersecting forms of discrimination. Future research is needed to investigate the intersection between obstetric ableism and obstetric racism.

Prior research has found that cissexist (anti-transgender) attitudes are prevalent among perinatal health care providers [88]. While neither of the two transgender participants in our study discussed intersections between disability and gender identity at length, the respectful labour and delivery care Jordan received as a disabled person was positively impacted by providers who were also knowledgeable and respectful of gender, as demonstrated by their attention to preferred names and pronouns. This example illustrates how respectful, disability-affirming labour and delivery care must also be gender-affirming and respectful of gender identity. Future studies should further examine the intersectional labour and delivery care experiences of transgender people with disabilities.

Many participants in our study articulated both positive and negative experiences of labour and delivery care that we interpret as exemplary of disability injustice and disability justice. While some of these experiences were with different providers in different facilities and during different birth experiences (e.g., Jasmine, Maria, and Garima), many of these experiences were with different providers in the same facility during a singular birth experience (e.g., Johanna, Robyn, Shannon, and Siobhan). This indicates that labour and delivery care experiences are complex, multilayered, and inconsistent, and that perinatal health care systems can oscillate between the provision of care that is in line with both disability injustice and disability justice, often thwarted or facilitated by encounters with different health care providers. Although we did not interview participants’ care providers and lack information about whether their care practices were informed by disability-related training, studies have found that most OBs do not receive disability-related training [37] and most perinatal health care providers who do have disability-related training are driven by their own initiative to acquire these valuable skills [38]. Further research is required to investigate if providers who offer collaborative, respectful, and disability-affirming labour and delivery care have received disability-related training, or if they are similarly driven by their own initiative to provide inclusive labour and delivery care. Further research is also necessary to investigate if, like health care systems and facilities, providers can be similarly inconsistent in their provision of collaborative, respectful, and disability-affirming labour and delivery care, as well as what factors support or hinder providers to adopt such care practices.

The dominant frameworks for maternity care in Canada and the United States are patient- and family-centred [73, 74]. Our findings of disability injustice in labour and delivery care indicate that disabled people are not consistently receiving perinatal care that is patient- or family-centered. Our analysis suggests that programs and policy, including perinatal care guidelines, should promote collaborative, respectful, and disability-affirming care to facilitate collective access and promote disability justice for pregnant people with disabilities, including through shared decision-making between patients and providers as a collaborative, respectful, and disability-affirming process.

We also add to prior calls to improve the quality of pregnancy care for people with disabilities through integrated, interdisciplinary team approaches [72] and increased disability-related training of perinatal care providers [37, 89–91]. Improving pregnancy care for disabled people and scaling-up disability-related training for perinatal health providers must include people with disabilities as key collaborators. We recommend that disability-related training of perinatal care providers address ableism and intersectionality, and stress the importance of collaborative, respectful, and disability-affirming care, especially through shared decision-making in clinical practice. The development of care guidelines, programs, and policies relevant to pregnant people with disabilities should also incorporate the ethos of shared decision-making by valuing the disability expertise of lived experience and collaborating with disability communities.

## Conclusions

In this article, we analyze people with disabilities’ experiences with labour and delivery care through a disability justice lens, examining experiences of disability injustice and disability justice through collective access. We recommend strategies for facilitating collective access to labour and delivery care for people with disabilities, including through collaborative, respectful, and disability-affirming care that reimagines care-related decision-making as an interdependent process shared between patients and providers, rather than an independent choice. We suggest that facilitating collective access to labour and delivery care through collaborative, respectful, and disability-affirming care can promote disability justice and improve the quality of perinatal health care for pregnant people with disabilities.

## Electronic supplementary material

Below is the link to the electronic supplementary material.

Supplementary Material 1

## Data Availability

In accordance with ethics approvals, the data analysis is based on individual interviews that are only available to researchers involved in this study.

## Footnotes

1 Because of the small sample size and to ensure anonymization, we use the terminology “person or woman of colour” to refer to participants with various racial/ethnic identities (*n* = 10), including East/Southeast Asian (*n* = 3), Black/African (*n* = 2), Indigenous (*n* = 2), South American (*n* = 2), and Arab Jewish (*n* = 1).

2 Vaginal seeding is a practice of swabbing surgically born babies immediately after birth with the birthing person’s vaginal secretions [57].

3 We use the concept of capacity critically, and not as a synonym for able-bodiedness, following Jasbir K. Puar [58]. We acknowledge the entanglement of capacity and debility in disabled life experiences, and that modes of privilege including whiteness and economic mobility can facilitate bodily capacitation. Our analysis of the dismissal and recognition of pregnant people with disabilities’ capacities seeks to challenge ableist assumptions that disabled people experience what Puar refers to as the fiction of “pure debility” and the lack of capacity.

## Abbreviations

ASL: American Sign Language
IDD: Intellectual/developmental disabilities
MS: Multiple sclerosis
OB: Obstetrician
